# The role of social work practitioners and human service professionals in long-term disaster recovery after the 2016 Alberta wildfires in Canada

**DOI:** 10.1177/00208728241269680

**Published:** 2024-08-09

**Authors:** Julie L Drolet, Bonita Lewin, Kamal Khatiwada, Evalyna Bogdan, Elladee Windsor

**Affiliations:** University of Calgary, Canada; The City of Calgary, Canada; University of Calgary, Canada; York University, Canada; University of Calgary, Canada

**Keywords:** Alberta wildfire, disaster recovery, disasters, human service professionals, social work, social work practitioners

## Abstract

The 2016 Alberta wildfires resulted in widespread destruction of property and displacement of residents. Research aimed to identify the roles and responsibilities of social work practitioners and human service professionals in long-term disaster recovery. This article presents the findings from interviews, focus groups, and a survey with a total of 140 participants in Alberta, Canada. Implications for disaster social work planning, and response and recovery efforts in Canada and internationally, will inform the development of programs and policies to support and make visible the contribution of social workers and human service professionals in long-term disaster recovery.

## Introduction

Social work practitioners and human service professionals play a crucial role in long-term disaster recovery efforts (Drolet et al., 2021; Bogdan et al., 2022). However, attempts to understand their professional role in disaster-affected communities have focused largely on the delivery of short-term relief services (Public Safety Canada, 2019). There is limited research that has examined the role of social work practitioners and human service professionals working in long-term disaster recovery in Canada. This research was conducted to identify the roles and responsibilities of social work practitioners and human service professionals in long-term disaster recovery after the 2016 wildfires in Fort McMurray, Alberta. The study objectives were: (1) to better understand the challenges experienced by social work practitioners and human service professionals engaged in long-term disaster recovery in Alberta; (2) to identify supportive social service practices and strategies for long-term disaster recovery in communities; and (3) to articulate a disaster social work professional practice framework at the local community level. This article will also highlight an outcome of the study, the creation of the Social Work and Disaster (SWAD) Network, which aims to build capacity, exchange resources, and create awareness of the role of social workers and human service professionals in disaster contexts.

## Context

The 2016 Alberta wildfires resulted in devastating human, economic, and environmental impacts. The Alberta Government declared a provincial state of emergency and issued a mandatory evacuation order for more than 80,000 people in the Regional Municipality of Wood Buffalo and Fort McMurray area (Schmidt, 2016). The most heavily fire-damaged neighborhoods included Abasand, Beacon Hill, Stone Creek, Wood Buffalo, and the portion of Waterways that lies above the flood hazard zone (Regional Municipality of Wood Buffalo, 2017). Wildfire crews from across Canada, the United States, South Africa, and Mexico supported the efforts in Alberta.

To meet the immediate needs of the evacuees, the Government of Alberta (2016a) provided emergency financial assistance in the form of pre-loaded debit cards of $1250 per adult and $500 per dependent. It is estimated that more than 48,000 wildfire relief debit cards were distributed in Alberta, totaling $96 million (Government of Alberta, 2016b). The Insurance Bureau of Canada (2023) reported that the Fort McMurray wildfire cost $5.4 billion, the most expensive insured catastrophe in Canada’s history. Before 1995, only three disasters in Canadian history had exceeded $500 million in losses (adjusted 2010 dollars). However, since 2010, $1-billion disasters have become an annual occurrence. Cumulative disaster losses have cost Canadians over $2 billion annually over the last decade (Cryderman, 2016). The top two costliest disasters in Canadian history (Cryderman, 2016) have occurred in Alberta, with the 2013 Alberta floods and the 2016 Alberta wildfires.

Alongside emergency financial aid, evacuees received access to counseling services and support from community programs. Funding was also provided to municipalities for ecological restoration efforts. This holistic approach aimed to alleviate the multifaceted impacts of disasters, recognizing the importance of addressing not only financial concerns but also the social, health, and environmental well-being of affected individuals and communities. The wildfires in Alberta underscored the interconnectedness of human, ecological, and economic factors in disaster resilience. Consequently, disaster risk reduction and resilience building have emerged as top priorities for municipal and federal governments, emphasizing a comprehensive approach to emergency management that integrates social, ecological, and economic considerations (Alberta Emergency Management Agency, 2014; Public Safety Canada, 2009). Disaster prevention and mitigation is a key focus in Canada’s emergency management efforts using a risk-based and all-hazards approach (Public Safety Canada, 2022) and disasters illustrate the complex interrelationships between vulnerability, risk, and resilience (Turnbull et al., 2013).

## Literature review

Disasters create crises that affect large populations and initially call for disaster management and emergency rescue operations to address the event (Roberts and Ottens, 2005). According to the United Nations Office for Disaster Risk Reduction (UNDRR) (2022), a disaster is defined as

‘a serious disruption of the functioning of a community or a society at any scale due to hazardous events interacting with conditions of exposure, vulnerability and capacity, leading to one or more of the following: human, material, economic, or environmental losses and impacts’ (para 1).

It is recognized that some disasters are exacerbated by climate change that is contributing to more frequent and intense natural hazards and extreme events, including wildfires (Intergovernmental Panel on Climate Change (IPCC), 2022). Apart from the instant impacts, disasters can have long-term impacts on individuals, families, and communities.

Climate change is increasing the severity and frequency of natural-hazard disasters such as floods, wildfires, storms, and droughts. New findings from the Intergovernmental Panel on Climate Change (IPCC) (2022) report indicate that the impacts of climate change are already being felt and that these impacts are expected to intensify unless immediate and substantial action is taken. Climate change adds a new dimension to community risks and exacerbates vulnerability and social and economic inequities, particularly for vulnerable and marginalized communities (Barbier and Hochard, 2018). ‘While the poor and marginalized contribute the least to climate change, they are also the most likely to be harmed by it and they have the fewest resources to cope with it’ (United Nations Research Institute for Social Development (UNRISD), 2022: 97), presenting a ‘double injustice’ (UNRISD, 2022: 97) at various levels that include income, race/ethnicity, citizenship status, employment status, gender identity and sexual orientation, among others. Forced displacement and migration due to disasters and environmental degradation has been on a rise in the last decade (United Nations Development Programme, 2014; Wahlström, 2015).

Social work practitioners and human service professionals are increasingly involved in long-term disaster recovery. The social work profession has a long history of addressing the needs of vulnerable populations, including those who experience natural or man-made disasters (Bliss and Meehan, 2008; Bogdan et al., 2022). However, it is becoming increasingly challenging to assess and respond to complex climate change risks. Complex climate change risk refers to situations in which multiple risks and uncertainties interact, creating a challenging environment for decision-making (Simpson et al., 2021). Complex risks result from multiple climate hazards occurring concurrently and from multiple risks interacting, compounding overall risk and resulting in risks transmitting through interconnected systems and across regions (IPCC, 2022). Complex climate change risk necessitates identifying and making visible the roles, values, and expertise of individuals and groups involved in disaster contexts. In Canada, and internationally, it is important to consider the contributions of social work practitioners and human service professionals in supporting disaster-affected communities through the provision of effective and compassionate services and programs at various levels.

Advocacy plays a crucial role in all phases of a disaster, from mitigation and preparedness to response and recovery (International Federation of Red Cross IFRC and Red Crescent Societies (RCS), 2009). In the recovery phase, advocacy efforts focus on promoting long-term recovery and rebuilding efforts that are inclusive, sustainable, and responsive to the needs of disaster-affected communities, including Fort McMurray, after the 2016 wildfires (Drolet et al., 2021; Bogdan et al., 2022). Advocates work to ensure that disaster-affected communities have access to essential services, such as housing, healthcare, and employment, and that their rights and needs are protected throughout the recovery process. Effective disaster response and recovery efforts aim to build individual and community resilience by providing emotional support, practical assistance, and access to resources (Drolet et al., 2021; Bogdan et al., 2022). This can help to mitigate the impact of disaster trauma and promote long-term recovery.

## Theoretical framework

The study adopted three theoretical perspectives: constructivism, psychosocial approach, and stepped care model. *Constructivism* is a framework that focuses on lived experience and the unique way individuals understand the world. It also focuses on recognizing and understanding the diverse and unique experiences of social workers and human services professionals in long-term disaster recovery. The *Psychosocial Approach* has two pillars: *psychosocial support* to improve well-being and minimize mental health issues (Inter-Agency Standing Committee (IASC), 2007) and *psychosocial capacity building*, which includes ‘intervention, provided by professional and non-professional people, both local and from the outside, that constitutes a multi-systemic, culturally grounded, empowerment- and resiliency-oriented approach designed to help individuals, families, social groups, and communities recover from a disaster’ (Miller, 2012: 191). Psychosocial capacity building aims to build on local capacities and resources (Miller, 2012). This can be used during a disaster response to assist individuals and communities in meeting their daily social and emotional needs (Alberta Health Services (AHS), 2021). The *Stepped Care Model* is a component of the psychosocial resilience framework that depicts the phases and timing of a disaster as well as the types of support required in each (AHS, 2021).

## Methodology

The research had three components with a total of 140 participants, which included individual interviews (n = 40), focus groups (n = 11), and a survey (n = 89). A purposive sampling approach was used to recruit interview participants from not-for-profit agencies, community organizations, social service agencies, and provincial and municipal government departments. Due to the onset of the COVID-19 pandemic and related health and safety protocols (e.g., social distancing), interviews, focus groups, and a survey were conducted virtually. The survey was developed in Survey Monkey with Likert-type scale, multiple choice, and open-ended questions, and was circulated throughout Alberta via the Alberta College of Social Workers, post-secondary social work faculties, and the Alberta Health Services (AHS) Psychosocial Disaster Network.

Recruiting social service professionals who assisted in the 2016 wildfire was challenging as some individuals who were impacted by the disaster had moved out of the community, left their workplace organization, or retired. Some were busy assisting their community recover from disasters that occurred before or after the wildfire, such as the pandemic, financial crisis, opioid crisis, and a flood in April 2020.

Between June 15, 2020, and August 31, 2020, individual interviews were conducted via telephone or Zoom with social workers and human service professionals who assisted in the recovery of the 2016 wildfire, and they were invited to participate in Zoom focus groups between March 25, 2021, and April 14, 2021. Of the 40 interview participants, 33 were social workers with professional designation. Findings based on the interviews alone were previously published (Drolet et al., 2021). Because of the low response to focus group invitations, the research team developed an online survey to capture perspectives and experiences. The survey was accessible to social workers and social work students between March 12, 2021, and April 14, 2021, in order to better understand the role and responsibilities of social workers before, during, and after a disaster with or without disaster experience. Of the 89 survey respondents, there were 20 survey respondents who answered yes to being impacted by the 2016 wildfire and these respondents were asked additional questions about their community’s recovery.

The data from the interviews and focus groups were audio recorded and transcribed prior to combining it with the survey data and then all data was thematically analyzed through the NVivo 12.0 software program. The analysis identified six themes that are discussed in the findings.

## Findings

The six themes identified from the analysis are (1) the role of social work in disasters; (2) understanding disaster trauma and resilience; (3) intersections that challenge disaster recovery; (4) social work advocacy in disaster phases; (5) importance of wellness and well-being of practitioners in disaster response and recovery; and (6) the need to build disaster skills for social workers through professional development. The thematic findings are elaborated on below.

### The role of social work in disasters

Survey responses indicated that support is required for individuals, families, and communities who have experienced a disaster. Specifically, 67% of the survey respondents indicated that they were personally impacted by a disaster and required services to support them in responding to the disaster and 50% stated they required recovery services and programs after the disaster. It was identified that many of the roles and skills social workers offer to individuals, families, and communities before, during, and after a disaster are the same that they use during non-disaster times in their community. One interview participant stated ‘I think we need to promote that social work is a valuable resource in a disaster, and in fact, in my opinion it is critical–absolutely critical’. Participants identified the roles and skills that they used during Alberta disasters as assessments, advocacy, community relationships and knowledge, counseling, crisis intervention, leadership, and program development.

Participants mentioned that the experience in completing community and individual assessments supported emergency management professionals in triaging emergent needs and the needs of individuals, families, and the most vulnerable in the impacted community. Individuals who were experiencing oppression or injustices in the community prior to the disaster were identified by study participants as vulnerable in the disaster context. For example, one focus group participant explained that vulnerable populations included ‘the people that really need these services, we’re talking about the new immigrants . . . the homeless on the street, you know people that really need to assess these services’.

Social workers were advocating for the emergent needs of individuals and their community to ensure equity for all who were impacted, as one interviewee stated ‘trying to advocate for more if needed and it was [on a] case by case basis’. Participants indicated how important their community relationships and knowledge were during and after the disaster as it supported emergency management professionals in learning about the challenges the community was facing and what current supports and services were available in the community. A focus group participant offered ‘you know the community agencies coming together really are going to be dealing with the recovery after an event’. Further, an interviewee commented:

‘How critical it is in that recovery piece in community . . . to build relationships, so I think sometimes place[s] that already had an existing relationship with clients, community members, they are much more able to support people and help people, rather than agencies parachuting in after a disaster’.

Participants who were counselors recalled that they provided an empathetic and non-judgmental response to impacted individuals and families. A focus group participant explained that individuals had complex needs and some were experiencing mental health stressors including grief and loss ‘you’re looking at a psychological effect, you’re looking at depression, you’re looking at somebody that is going out of their comfort zone’. Crisis intervention skills were identified as crucial during disasters as it provided short-term support during the response.

Some participants identified having leadership skills that were never utilized and that these skills could have been used to develop equitable and accessible programs for their community. There were roles participants did during and after the 2016 wildfire that were identified as roles normally performed by emergency management professionals in their community. These roles included writing business continuity plans, collaborating with community members and other organizations on mitigation strategies, preparing individuals for future disasters, training first responders and community members on the psychosocial needs of individuals during a disaster, and creating plans and programs to support families and individuals during and after disasters. An interviewee noted:

‘My role at a provincial policy level there certainly were roles for prevention and post-prevention, and in terms of kind of resource allocation and even policy creation, and program development to ensure there is kind of an opportunity to support those areas’.

The study found that the role of social workers in disasters was often unrecognized and undervalued by emergency management professionals despite the many roles and responsibilities performed during and after the 2016 Alberta wildfire, as well as other disaster events such as the 2013 Southern Alberta Floods and 2011 Slave Lake Alberta wildfire. A participant explained why emergency management professionals and first responders (fire or police) may not call upon social workers or social service professionals in disaster response or recovery:

‘A typical disaster response from a first responder point of view, [they] are very much looking at the infrastructure and the hard services that first responder, the first responder kind of piece, and I think the social work pieces really look at that social work or that psychosocial piece and supporting people where they’re at to figure out, to navigate the system which is often can be very challenging, especially for people with vulnerabilities’. (Focus group participant)

There was a lack of acknowledgment that social workers were involved in the disaster response and recovery, as expressed by an interview participant: ‘The first responders get huge accolades, you know, wonderful stuff, but people forget that it’s guys like me that keep them working and nobody mentions the social worker’. Being undervalued for their contributions in the disaster was discouraging for participants as they continued to support the community in recovery.

### Understanding disaster trauma and resilience

Participants identified using social work theories and concepts of person-centered, strength-based, solution-focused, person-in-environment, and an anti-oppressive lens to support individuals and communities with a focus on trauma informed care and resiliency. An interviewee said: ‘understanding that it’s the responses and the effect of disasters on clients . . . has ripple effects’. A focus group participant shared that ‘a doctor challenged me on wanting to support a firefighter . . . and the response was well that was that fire was like eight months ago, like shouldn’t people be over that by now’.

A person-centered approach was mentioned in the interviews, focus groups, and survey as a key theory to use during disasters, as it allowed the social workers to focus on the unique needs, experiences, and trauma of the impacted individuals, families, and community. For example:

‘Person-centred work–solution-focused, person-centered–because what I think is hard for some people to realize if they have never been in a disaster situation is that everyone responds to it very differently, and so your work really has to be person-centered, and it is not going to be a one size fits all support that fits for everyone’. (Interviewee)

Strength-based and solution-focused concepts were identified as the community was re-establishing itself and creating connection opportunities for individuals and families after the wildfires. The person-in-environment was observed as practitioners acknowledged that individuals were imbedded in their environment with personal and community connections after the wildfires. An interviewee remarked ‘So, ensuring that people have connections in community so that they are not isolated’.

Study participants identified that social workers are trained to deliver anti-colonial and anti-racist services to individuals, families, and communities. They thought that knowledge could be shared with emergency management to assist them in creating emergency preparedness, response, and recovery services, programs, and plans that were more equitable and accessible:

‘I think a real anti-colonizing, anti-racist perspective is also really important. I recall a few instances of people trying to explain their story or explain their particular living situation and I think if people don’t understand some of that history around colonization and the impacts of colonization those stories don’t necessarily make sense, and there is the opportunity or risk of further colonizing people during disaster’. (Interviewee)

Participants discussed the importance of having a trauma informed care approach when supporting individuals, families, and communities during and after a disaster. An interviewee explained understanding trauma during a disaster as:

‘Recognizing that the family has been impacted not only by their personal circumstances, but also the community and all the individuals within the community have also been impacted and the compound trauma recognizing that nothing happens within itself that it’s not a standalone event that it is compounding from other events, whether it be natural disasters or personal mental health’. (Interviewee)

Comments were made about the importance of being aware that individuals and families may have generational or personal trauma that could create adverse emotional reactions during a disaster. One focus group participant stated that individuals impacted by a disaster might be ‘triggered by [their] residential school experience’. Study participants identified that individuals, families, and communities may also experience long-term trauma after a disaster.

Resiliency was identified by some participants after they observed individuals connecting with their informal supports and when community members voiced their concerns about recovery efforts in the community. Some participants mentioned they observed resiliency in the way the community responded to other disasters (e.g., the COVID-19 pandemic and flood event) after the 2016 wildfires. Several participants mentioned that they were resilient during and after the wildfires and one focus group participant said ‘I was more resilient than expected’.

### Intersections that challenge disaster recovery

**Figure 1.**
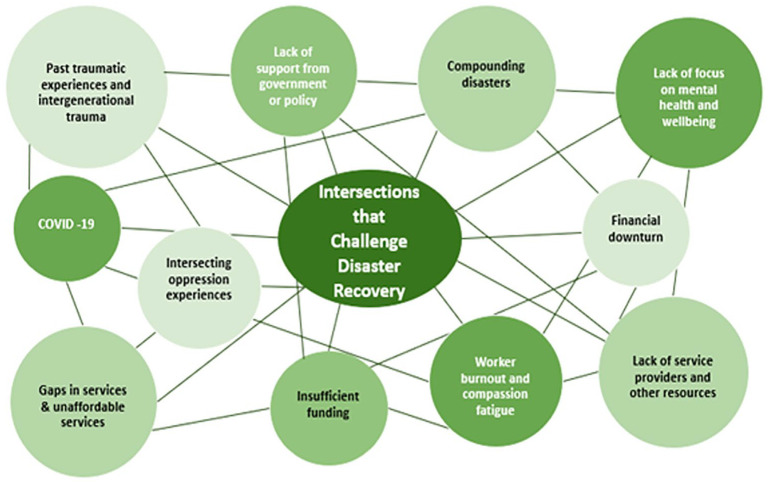
Complex risks and intersections after the 2016 Alberta wildfires.

The study found that participants described complex risks and multiple influences that affected disaster recovery post-wildfire. Displayed in Figure 1 is the variety of intersections that challenged the recovery of the individuals, community, and families after the 2016 wildfires.

The economic and financial downturn in Alberta was identified by many study participants as a key consideration both prior to and after the 2016 wildfire:

‘If you’re looking at it through a compound trauma lens, we’re nowhere near where we need to be we’re, we’re, barely we’re just treading water . . . because of the economic downturn has significantly impacted us to the point where we’re, we’re struggling, and we’ve lost so much and financially. People are going bankrupt and just leaving their houses behind and moving on that’s the truth of our community, right now, which impacts their mental health in spiral, spiral, spiral’. (Focus group participant)

Four years after the wildfire, in 2020, the COVID-19 pandemic severely disrupted health and social services and the community also experienced a flood in April 2020. Social workers identified that these compounding disasters impacted individual and community recovery. Some focus group participants shared that because of the compounding disasters, they decided to leave the community, for example:

‘You cannot look at them in isolation, they are compounding. . . one affects the other and it absolutely has an impact on the individual and how they’re going to cope, and even if they had resiliency before the blows that the community has been taking, the financial downturn is just as significant, if not more significant than some of the natural disasters’. (Focus group participant)

Other intersections identified were insufficient long-term funding opportunities and a lack of support from government bodies. A focus group participant stated that ‘long-term mental health impacts come later’, and participants agreed that 3-year funding grants were insufficient to support and sustain recovery. There was discussion on how the government could support recovery by financially supporting pre-existing services in the community and supporting families who were having financial challenges in obtaining compensation from their insurance claims. Some participants spoke about government eligibility criteria that was viewed as oppressive because it prohibited some individuals and families in obtaining financial support:

‘I think oftentimes agencies . . . have eligibility criteria or intake criteria right, and oftentimes, that is, those criteria, maybe in a disaster circumstance needing to be bent a little bit, right in order to ensure that services can be accessed by those who need them’. (Focus group participant)

### Social work advocacy in disaster phases

In reflecting on their experiences in the 2016 wildfires and other Alberta disasters, study participants identified advocacy work as a critical component of social work practice at the micro, mezzo, and macro levels in all disaster phases (mitigation, preparedness, response, and recovery). Participants advocated for social workers to be involved in disaster planning because they identified that they have community knowledge that can enhance the community’s ability to mitigate disasters, prepare for a disaster, respond to, and recover from a disaster. Because social workers and human service professionals have long-standing relationships with individuals, families and the community prior to a disaster event, these existing relationships can be used to support a community’s recovery.

Advocacy at the micro level focused on obtaining equitable and accessible services to meet the unique and complex needs of individuals and families. One interview participant described their advocacy as a way to:

‘Ensure that our clients were adequately taken care of and properly treated, and particularly to the hotels who might not want to accommodate them because of the way that they look, or because of stereotypes, so we had to advocate extra for clients to make sure they had the resources they needed’. (Interviewee)

Participants advocated to insurance companies to support their clients respectfully and ensure they were provided the financial support outlined in individual insurance policies as one interview participant identified ‘trying to keep my clients housed during a time of turmoil, trying to keep them in housing and making sure they were a priority’.

There were gaps in services identified by participants as they tried to facilitate individual and family referrals to current health and social services and programs. An interviewee explained ‘being able to advocate for whatever service or program, it is that you’re trying to get that that client into’.

Advocacy at the mezzo level focused on community programs and interprofessional networks to support recovery. An interview participant exclaimed: ‘we advocated for a program that included things like dance in the morning to help regulate kids’. Participants saw a need to build and enhance community relationships with schools, interprofessional networks, and social service organizations. One interviewee described supporting other non-profit organizations in developing business continuity programs: ‘we have been doing some advocacy around the need to support non-profits in their ability to both internally respond and externally respond to disaster situations by having strong business continuity programs’. Relatedly, recovery support needed to be inclusive, accessible, and culturally appropriate. An interview participant shared the need for supports in recovery:

‘Communities and certainly individuals and families as a part of those communities, really need sort of, basic psychosocial support they need to understand what their experiences, they need to understand and have that experience validated and normalized and certainly be attached to supports in the community’. (Interviewee)

At the macro level, advocacy centered on recovery funding models and government policy. Participants stated that individuals and community recovery programs required financial supports for a longer period than what was made available in their community. For example, programs that were developed to support individuals recover from the wildfire were closed due to the lack of financial resources to sustain programs because of the limited duration of wildfire recovery grants.

‘I would say there’s lots of programs and services in our region now whether they’re funded any more via recovery fire recovery is a different thing, I think, of course, we used to have a lot more because post wildfire you know, there was a mass influx of dollars, and this is the year that last year and this year, most of the fire recovery dollars have reached their sort of end of contracts so there’s only a few little programs sprinkled around that are specifically funded via fire recovery’. (Focus Group Participant)

It was identified that social workers were educating policy makers on the need for government policies on supporting the psychosocial and socio-economic needs after the wildfire as those needs were still evident in the community.

‘I am thinking about this from a macro level because that is what my experience was at the fire level . . . working with the Department of Environment and Finance, and that sort of thing, so we were sort of working more with the bureaucrats and educating them . . . you are kind of educating accountants around trauma and the need for longer term resources, around the trauma of lining up at the Butterdome [for services] and what that is like’. (Interviewee)

The survey identified a disparity that was occurring during the COVID-19 pandemic due to the lack of technology (e.g., lack of electronic records and few laptops) to stay connected with family, community, and social programs. A survey respondent identified the need for appropriate technology as a ‘basic human right’ as virtual connections were expected, and there is a need to advocate for adequate Wi-Fi and Internet connectivity in communities:

‘My field is community development, focused on bringing people together at a neighbourhood level. With our connections becoming primarily virtual, it has become difficult to connect with those who don’t have appropriate technology of access to the internet. Access to technology and the internet has become a basic human right and more advocacy is needed (especially as a means of disaster preparedness)’. (Survey respondent)

### Importance of wellness and well-being of practitioners in disaster response and recovery

Study participants identified the need for wellness and self-care strategies and dialogue within their organizations to support the emotional toll disasters have on first responders (police, fire, medical) and social workers. Participants discussed the need to address stigma in relation to wellness strategies in organizations as they observed a perceived weakness when someone takes time for themselves, as other staff would experience an increased workload.

Some participants felt they could not take time to care for themselves as their organization was experiencing high staff turnover. A focus group participant said: ‘you know I think it’s hard because there’s so much turnover and people leave’. Participants shared that social workers are aware of vicarious or secondhand trauma but forget to care for themselves, as one focus group participant shared: ‘even though we have been trained that we are in a risk, if we are not taking care of ourselves’.

Several participants spoke about feeling fatigued and tired in dealing with the COVID-19 pandemic while still recovering from the wildfire. A survey respondent explained that ‘it has made me feel isolated and sad, depressed’, and another survey respondent stated that ‘working during COVID is very stressful and at times can take a lot of energy, working and attending classes part-time takes a lot of energy in no-COVID times – during COVID it can be exhausting’. The survey found that 82% of the respondents identified that COVID-19 impacted their mental health and work-life balance.

Participants discussed experiencing caregiver burnout because they were personally impacted by the wildfire and responsible to care for a loved one during the COVID-19 pandemic. Compassion fatigue was mentioned by several participants who continued to support individuals who were impacted by the wildfire during a period when their workload intensified due to the COVID-19 pandemic and after the 2020 flood. A focus group participant shared how they were overwhelmed by the 2020 flood impacts:

‘You wax and wane between being like, just so overwhelmed with it, that it’s just like, it can feel all consuming, I literally have moments of like how much more can we take, and how much more as a social worker can I give, when you’re just coming up against it, seems like our community just can’t get a break’. (Focus group participant)

Several participants stated that their well-being was paramount for them to offer trauma informed service and assist in building a resilient community post-wildfire. Social workers and human service professionals identified the need to take care of themselves and recognize when they need to recharge. An interviewee called it ‘part of your professional responsibility’ and another interviewee explained ‘I incorporate a lot of self-care into my own practice to make sure I am in a good place to help clients’.

There was an overwhelming need identified by study participants for a disaster social work peer support program as a way to connect and to learn from other social workers. One interview participant offered ‘have peers you can reach out to as a support system’. It was identified that such a program would support social workers without disaster experience or training.

### The need to build disaster skills for social workers through professional development

Participants indicated that they were personally not prepared for the 2016 wildfire and the survey results found that 45% of respondents indicated that they were not prepared when they experienced a disaster. The focus groups identified the important learning from disaster experiences, and one focus group participant offered the following: ‘I think due to COVID, due to the flood, we’ve had to change the way we do things for example being on Zoom right now it’s, it’s exhausting because of the Zoom fatigue, but it also helps us connect’.

Interview and focus group participants explained that social workers required disaster training and stated how important it was to have knowledge about disasters and emergency management to support their community during and after a disaster. Several participants suggested that social workers take Incident Command System (ICS) training to provide social workers with an understanding of the language used by emergency management officers and knowledge of the chain of command in a disaster. One focus group participant suggested: ‘they [emergency managers] operate from a very specific system called the ICS system . . . to offer a disaster perspective . . . we can speak the same language’.

Participants who had disaster experience identified a variety of trauma, psychosocial and community development trainings that could support individuals and communities’ post-disaster. The professional development and training opportunities identified were: Psychosocial First Aid, Heart Math, Post-traumatic Stress, Loss and Grief, Critical Incident Stress Management, Eye Movement Desensitization and Reprocessing (EMDR), Mental Health First Aid, and Skills for Psychological Recovery. Learning from each other was another approach discussed by study participants as a way to learn from social workers who have disaster experience. An interviewee stated ‘after the [wild]fire I went to Calgary and did a two-day workshop on disaster response and recovery, and so that helped, just seeing how different disasters around the world are like’. One focus group participant identified that ‘if we hadn’t had the flood experience or that, that opportunity to really get more organized, I think we would be making it up every time’.

The interview and focus group participants agreed that there was a need for social work institutions to prepare social workers to support their community after a disaster. Many participants reflected on the numerous disaster events locally and internationally, and that there were more destructive disasters occurring. One suggestion was to offer disaster training in the post-secondary social work community development curriculum as disasters impact the whole community. Some participants were aware of multiple disaster training available as professional development opportunities, and some were not aware, as stated by a survey respondent ‘I don’t actually know what all of these are’.

## Discussion

The study findings demonstrate that social workers are involved in all phases of disasters and serve a critical role in building resilient individuals, families, and communities before, during, and after a disaster. Social workers and human service professionals advocate structural (macro level) change and shape policy response and mitigation planning. The findings also show that social workers are involved at the micro and mezzo levels: preparing individuals and communities for future disasters, writing business continuity plans, collaborating with community members and other organizations on mitigation strategies, training first responders and community members on the psychosocial needs of individuals during a disaster, and creating plans and programs to support individuals and families during and after disasters. However, these important contributions from social workers are often overlooked by emergency management professionals in Canada, which is also noted by Bogdan et al. (2022) who argue that more interdisciplinary collaboration is needed. Similarly, in the United States, Cederbaum et al. (2022) found that social work is part of the essential workforce historically and throughout the COVID-19 pandemic, yet lack recognition. Advocating for social workers to have a visible presence in disaster contexts requires practitioners to work together, become trained in emergency management, build awareness of climate change, and promote their many contributions. The unique contributions of social workers and human service professionals need to be acknowledged to improve disaster mitigation and preparedness to response and recovery.

Complex risks and intersections impacted disaster recovery at the community level and demonstrate the need for long-term sustainable and equitable recovery plans that include the provision of social services and programs, as well as psychosocial and wellness strategies (IPCC, 2022). In Alberta, the economic and social crisis impacts are closely related as economic crises affect employment, household incomes and consumption, and the provision of social services (Kingsley et al., 2021). The study findings provide evidence to suggest that disaster recovery is not a linear or short-term process. Numerous intersections and complex risks impact disaster recovery as seen in the aftermath of the 2016 Alberta wildfires. Disaster recovery must be linked to social development plans and objectives to foster resiliency at all levels, with sustained financial resources to support the delivery of long-term health and social services. The study found that many roles and skills needed before, during, and after a disaster were similar as during non-disaster times. Social workers and human service professionals have important knowledge and skills to contribute such as assessments, advocacy, community relationships and knowledge, counseling, crisis intervention, leadership, and program development to help achieve responses across the diversity of needs, build resilience, and undertake effective mitigation and recovery planning.

To build a resilient community post-disaster, it is critical for social workers, and the organizations in which they work, to recognize the importance of taking care of themselves as they support the community and impacted individuals. The study found that social work support could be strengthened through a SWAD Network, an outcome of this research project. The SWAD Network aims to facilitate information exchange, networking, and relationship building. The network is offering professional development opportunities and training to SWAD members in the context of disasters. The SWAD network aims to build capacity and create awareness of the role of social workers in disaster contexts to contribute to building a more resilient, inclusive, and sustainable society while reducing inequalities and vulnerabilities in Alberta.

## Conclusion

Alberta has experienced several major disasters in recent years: the 2013 floods, the 2016 wildfires, and the current COVID-19 pandemic. The literature and study findings clearly show that social work practitioners and human service professionals can play an important role in disaster contexts. However, there is a need to make visible roles and build capacity for professionals working in human services. This study aimed to better understand the roles of social work practitioners and human service professionals in long-term disaster recovery in the aftermath of the 2016 Alberta wildfire. The findings show that there needs to be clear role for social work practitioners and human service professionals in long-term disaster recovery, more sustainable long-term services, and wellness training as well as capacity building for social workers and human service practitioners. As a result of these research findings, the SWAD network was created to build capacity and create awareness of the role of social workers in disaster contexts to contribute to building a more resilient, inclusive, and sustainable society while reducing inequalities and vulnerabilities in Alberta.
